# We must rigorously follow basic infection control procedures to protect our healthcare workers from SARS-CoV-2

**DOI:** 10.1017/ice.2020.394

**Published:** 2020-08-03

**Authors:** Samuel W. Dooley, Thomas R. Frieden

**Affiliations:** Resolve to Save Lives, an initiative of Vital Strategies, New York, NY, USA

## Abstract

Because severe acute respiratory coronavirus virus 2 (SARS-CoV-2) spreads easily and healthcare workers are at increased risk of both acquiring and transmitting infection, all healthcare facilities must rapidly and rigorously implement the full hierarchy of established infection controls: source control (removal or mitigation of infection sources), engineering and environmental controls, administrative controls, and personal protective equipment.

As the severe acute respiratory coronavirus virus 2 (SARS-CoV-2) pandemic rages, healthcare facilities are inundated by patients with coronavirus disease 2019 (COVID-19). Many healthcare workers (HCWs) are being infected, and many are dying. Improving HCW protection is critical to preserve their health and to continue essential healthcare services. Healthcare facilities must rapidly and rigorously implement the full hierarchy of comprehensive infection control measures.^1^

SARS-CoV-2 spreads easily and can be transmitted by persons with asymptomatic or presymptomatic infection.^2,3^ Infection control planning must address all major modes of person-to-person transmission of SARS-CoV-2: (1) large-particle respiratory droplet transmission, when persons with COVID-19 cough; (2) contact transmission, such as by touching persons who have COVID-19 or surfaces or objects they have touched or coughed on; and (3) airborne transmission during aerosol-generating activities or procedures (eg, bronchoscopy). Aerosolization may be more common than previously recognized. The dichotomy between droplet and aerosols is false: droplet size is a continuous variable, and the combination of the volume and size of droplet generation and ventilation will determine the duration and distance within which transmission may occur.^4^

The hierarchy of controls includes (1) source control (removal or mitigation of the source of infection), (2) engineering and environmental controls, (3) administrative controls, and (4) personal protective equipment.

Source controls, engineering and environmental controls, and administrative controls are first-line interventions. Personal protective equipment (PPE) adds another layer of protection and is essential when the other approaches cannot guarantee complete protection. PPE is effective only when used consistently and correctly as a supplement, not a substitute, for the other approaches.

## Source control

The source of infection is a person with SARS-CoV-2 infection. The number of patients attending healthcare facilities should be minimized by (1) advising persons with mild symptoms to be tested safely and then isolate, monitor their condition, and only seek in-person care if symptoms worsen; and (2) using telemedicine to provide care for patients whose medical needs can be addressed remotely. For patients requiring in-person care, transmission risk can be mitigated in the following ways: (1) All persons must wear face masks when on the premises. (2) All persons with COVID-19 symptoms must be identified before they enter the facility and they should be separated from other patients. (3) Persons who are coughing must be moved to a well-ventilated area separate from other patients. (4) Persons admitted with known or suspected COVID-19 must be isolated in well-ventilated, single-person rooms with doors closed. (5) Infection prevention precautions must be implemented and maintained for 10 days after symptom onset and 3 days after recovery (ie, resolution of fever without use of fever-reducing medications and improvement in respiratory symptoms),^5^ and if there are not enough single-person rooms, patients with confirmed COVID-19 may be housed in cohorts in shared areas, according to CDC guidance, And (6) for persons with known or suspected COVID-19, aerosol-generating procedures must be performed in airborne-infection isolation rooms.

## Engineering and environmental controls

For potentially airborne infections, engineering controls consist of measures that: (1) create directional air flow to move air away from uninfected persons; (2) dilute the air to reduce the concentration of airborne infectious particles; (3) remove such particles from the air by high-efficiency particulate air (HEPA) filtration or effective UV light installation; and (4) prevent recirculation of potentially contaminated air. Some examples include enhanced ventilation in waiting areas, performing aerosol-generating procedures in airborne infection isolation rooms or the equivalent, and use of droplet and aerosol capture devices during aerosol-generating procedures.^6^

Examples of environmental controls include (1) plexiglass partitions between patients and staff in triage areas and (2) meticulously cleaning and disinfecting potentially contaminated surfaces and objects in all parts of the facility.

## Administrative controls

Administrative controls relate to the development, implementation, and enforcement of infection control policies and procedures (Box 1). These must be written clearly, easily understood by all staff, and posted conspicuously throughout the facility. To be effective, administrative controls must include: (1) training of all HCWs, at all levels, with role-specific practical instruction (e.g., checklists); (2) reinforcement of training to provide frequent reminders of key points; (3) quality control to assess adherence to policies and procedures; and (4) strict enforcement.

Box 1. Examples of Administrative Controls to Reduce SAR-CoV-2 Spread in Facilities

1. Establish and enforce strict, universal triage procedures
2. Require and enforce frequent, meticulous disinfection of surfaces that may be contaminated with SARS-CoV-2
3. Require and enforce frequent hand washing and sanitizing by staff (and patients)
4. Require that all patients wear face masks correctly and continuously, and enforce this requirement except in the case of respiratory compromise
5. Require and enforce separation of persons who may be contagious from all other persons (isolation) and periodically assess whether this is being done consistently and correctly
6. Require and enforce stringent precautions for aerosol-generating procedures while ensuring that only essential procedures are performed and that full precautions are taken whether or not the patient is suspected of having COVID-19
7. Ensure appropriate use of face masks, respirators, and other PPE
8. Restrict hospital visitors
9. Restrict HCWs with symptoms consistent with COVID-19 from working, while ensuring that they have paid sick leave
10. Require HCWs to wear at least surgical masks, and N95 respirators if available, for all interactions within 2 m (6 feet) of patients and staff

## Personal protective equipment

Personal protective equipment may include face masks, respirators, face shields, goggles, gowns, and gloves.

Face masks are loose-fitting, single-use paper or fabric devices covering at a minimum the wearer’s nose and mouth (eg, surgical face masks). They protect against transmission by large-particle droplets and sprays or splashes but not against aerosolized particle inhalation. For patients, face masks provide source control to prevent transmission of infection. For HCWs, face masks (ideally N95 respirators) prevent droplets expelled by patients from landing on their nasal or oral mucosa or from being inhaled. Face shields can further reduce risk by providing eye protection and blocking large particles.

Respirators cover at least the wearer’s nose and mouth and reduce the risk for inhaling aerosolized particles. They protect against airborne pathogens (eg, tuberculosis, measles) and are used during aerosol-generating procedures. Most respirators used in healthcare settings are filtering facepiece respirators, which use fine filter material to prevent the inhalation of aerosolized particles.

Single-use N95 filtering face-piece respirators are widely used in healthcare facilities. Reusable elastomeric half–face-piece respirators are an option but are rarely used due to potential interference with communication and user unfamiliarity. Both provide similar protection, require tight facial seal, and require fit-testing and training before use. Another option is reusable powered air-purifying respirators (PAPRs), typically loose-fitting hooded or helmeted devices with blowers that force air through HEPA filters for the wearer to breathe. PAPRs provide higher protection than N95 or elastomeric half-facepiece respirators, do not require fit testing, and can be used by people with facial hair, but they are costly and can interfere with communication and performance of duties.

## Face masks, respirators, and SARS-Cov-2

The CDC recommends that, when available, respirators be used rather than face masks for all HCWs entering areas where patients with known or suspected COVID-19 receive care, and eye protection should also be used in such situations.^7^ Respirators should be prioritized for persons present during aerosol-generating procedures if supplies are restricted; otherwise, respirators should be used by all who provide care to patients with known or suspected COVID-19. Use of reusable elastomeric half–face-piece N95 respirators or PAPRs could resolve supply issues, and these products can be safely cleaned by either wiping, or, after temporary removal of the filter, immersion.

Because many persons with asymptomatic COVID-19 are contagious, all HCWs should wear masks at all times in all areas of healthcare facilities.^1^ The rationale for this is 2-fold: (1) doing so may protect HCWs from being infected by patients or other HCWs with asymptomatic COVID-19; and (2) mask wearing may prevent HCWs with asymptomatic COVID-19 from infecting patients or other HCWs, including in nonclinical staff areas.

Although every healthcare facility must implement the full hierarchy of source, environmental or engineering controls, administrative controls, and PPE controls, the scale of this epidemic necessitates thinking beyond individual healthcare facilities. Especially in areas with many cases, most persons with known or suspected COVID-19 could ideally be channeled to designated facilities. Infection control procedures would still be needed in all facilities, but enhanced efforts could concentrate on COVID-19-designated facilities, and reusable PPE could be safely used, maintained, and disinfected. Critical supplies, equipment, and treatments could be allocated to designated facilities more efficiently. This would require participation by most or all hospitals in a geographic area, with centralized coordination, but might ultimately reduce the toll of this pandemic on patients, HCWs, and society.
